# Shared decision‐making with adults transitioning to long‐term care: A scoping review

**DOI:** 10.1111/opn.12518

**Published:** 2022-12-08

**Authors:** Caroline Egan, Corina Naughton, Maria Caples, Helen Mulcahy

**Affiliations:** ^1^ School of Nursing and Midwifery University College Cork Cork Ireland

**Keywords:** caregivers, long‐term care, older adults, scoping review, shared decision‐making, transitions

## Abstract

**Background:**

Transitions to long‐term care are challenging for individuals and often associated with a loss of autonomy. Positive experiences are noted, especially when decisions involve the individual in a person‐centred way which are respectful of the person's human rights. One approach which facilitates self‐determination during a transitional period is shared decision‐making, but there is a lack of clarity on the nature and extent of research evidence in this area.

**Objective:**

The purpose of this scoping review is to identify and document research related to shared decision‐making and transitioning to long‐term care.

**Methods:**

A comprehensive search in CINAHL, Medline and Psych‐info identified papers which included evidence of shared decision‐making during transitions to a long‐term care setting. The review following the JBI and PAGER framework for scoping reviews. Data were extracted, charted and analysed according to patterns, advances, gaps, research recommendations and evidence for practice.

**Results:**

Eighteen papers met the inclusion criteria. A body of knowledge was identified encompassing the pattern advancements in shared decision‐making during transitions to long‐term care, representing developments in both the evidence base and methodological approaches. Further patterns offer evidence of the facilitators and barriers experienced by the person, their families and the professional's involved.

**Conclusions:**

The evidence identified the complexity of such decision‐making with efforts to engage in shared decision‐making often constrained by the availability of resources, the skills of professionals and time. The findings recognise the need for partnership and person‐centred approaches to optimise transitions. The review demonstrates evidence of approaches that can inform future practice and research to support all adult populations who may be faced with a transitional decision to actively participate in decision‐making.

**Summary Statement of Implications for Practice**

**What does this research add to existing knowledge in gerontology?**

- The review identifies the type and level of international evidence exploring shared decision‐making with adults who are experiencing a transition to long‐term care.
- The findings demonstrate approaches and evidence that can be applied to influence future practice, research and policy to support populations who may be faced with a transition to actively participate in decision‐making.

**What are the implications of this new knowledge for nursing care with older people?**

- The review offers evidence of the facilitators and barriers to shared decision‐making which could assist nurses to support the older person in transitional decision‐making.
- Community nurses could play a key role in educating and engaging older adults in shared decision‐making.
- This review provides nurses with practice‐based approaches which could facilitate older adults with cognitive impairment engage in shared decision‐making.

**How could the findings be used to influence policy or practice or research or education?**

- The interdisciplinary focus of the evidence reflects all professionals including clinicians, researchers, policy makers, research commissioners and service providers who support older adult populations.
- The development of a workshop with user friendly resources could be used to educate nurses and other homecare workers supporting adults to engage in shared decision‐making.
- This review provides evidence which aligns with the United Nations sustainable developmental goals namely to reduce discrimination, inequality and promote inclusion of populations.

## 1 INTRODUCTION

Shared decision‐making (SDM) is described as a joint process whereby healthcare professionals work together with the person to reach a decision about their treatment and care (National Institute of Health and Care Excellence, 2021). SDM facilitates a partnership approach, in essence Elwyn et al. (2012) argues that SDM is dependent on a respect for the ethical principle of self‐determination, wherein healthcare professionals support the autonomy of the person to make decisions. There are many approaches to SDM with Bomhof‐Roordink et al. (2019) identifying 40 SDM models of which the key elements include making decisions, information exchange and facilitating choice. However, SDM can be challenging for some populations especially when decisions are presented to people following a health or care crisis (Bunn et al., 2018). One such challenging circumstance involves SDM with a person involved in a residential transition to LTC. Indeed, such transitional decision‐making are often more dynamic, complex and contextual than other treatment related decisions.

International figures report that between 1 and 5% of the world's population live permanently in a long‐term care (LTC) setting (World Health Organisation, 2022). The term LTC describes a variety of services including residential facilities designed to support a person's health and personal care needs for a period of time (National Institute on Ageing, 2017; Zimmerman & Sloane, 2007). The demand for LTC provision is predicted to increase due to population ageing, improved survivorship with long‐term conditions and societal changes within family structures (Organisation for Economic Co‐operation and Development, 2022), which may necessitate a transition to a residential LTC setting (Chyr et al., 2020; National Institute on Ageing, 2017). Each new resident will experience a transitional period described as a passage of time where the individual moves from one life phase, situation or status to another (Meleis, 2010, p. 11). Such transitions occur prior to, during and for a period of time after the relocation. Transitioning to a LTC setting is considered among the most significant and disruptive experiences for a person and their family.

Transitioning to LTC is not always associated with negative experiences; nonetheless, the majority of papers tend to focus on the negative aspects (Davison et al., 2019; Johnson & Bibbo, 2014). One such experience is the loss of autonomy (O'Neill et al., 2020; Paddock et al., 2019). Conversely, positive experiences were noted, especially when decisions involved the individual in a person‐centred way which were respectful of the person's right to self‐determine (Brownie et al., 2014; Gilbert et al., 2015; Regier & Parmelee, 2021; Richards, 2011). However, strategies which promote self‐determination by involving the person in decision‐making are often not prioritised during transitions into LTC (O'Neill et al., 2020). SDM is proposed as an approach to facilitate the person's involvement in such transitional decisions.

Despite an increased awareness and utilisation of SDM in health and social care, a preliminary search of existing systematic and scoping reviews identified 2 reviews. Initially, Gravolin et al. (2007) assessed the effectiveness of decision‐making support interventions delivered by professional staff on the outcomes for older adults facing the possibility of entering LTC. The second was a scoping review by Manthorpe and Martineau (2010) which sought to identify and analyse evidence on advocacy in relation to the decision to move to a LTC facility. Both reviews identified no evidence involving SDM during transitions to LTC. Presently, there is a lack of clarity on the nature and extent of research evidence on how SDM can be utilised by nurses and other professionals as an approach to facilitate the persons involvement in transitional decision‐making. Therefore, the current state of research and practice is still unclear which gave impetus for this review.

### 1.1 Aim and objectives

To identify and document the nature and extent of research evidence related to SDM and transitioning to LTC among adults.

Review objectives

- Describe the characteristics of evidence on SDM within the context of transition to LTC.
- Examine developments in SDM and how it is operationalised and evaluated.
- Identify the facilitators and barriers to SDM.

## 2 METHODS

It was recognised that the evidence may originate from a variety of scientific fields involving different methodological approaches. Therefore, a scoping review was chosen as an approach to evidence synthesis. This review was based on the Joanna Briggs Institute Manual for evidence synthesis (Peters et al., 2020). This framework was chosen as it details a set of steps to ensure a systematic approach using both the PICO and Prisma ScR checklist ensuring reproducibility of findings. The PAGER framework (Bradbury‐Jones et al., 2021) was also used to provide a structured approach that guided the reporting of this scoping review through the analysis of Patterns, Advances, Gaps, Evidence for practice and Research recommendations. The PCC mnemonic (population, concept and context) was used to identify the main concepts and inclusion criteria for the scoping review (Peters et al., 2020). The full details of inclusion criteria are outlined in Table 1.

**TABLE 1:** Inclusion criteria

PCC element	Definition/inclusion criteria
Population	Adult: A person aged 18 years and older who had relocated to a long‐term care facility or was considering a future relocation. Informal caregivers included family members or any individual who provided continuing care and support to an individual without financial reward. Formal caregivers involved paid staff including nurses, social workers, case managers, social carers, health care assistants and other allied health professionals who were involved in supporting a person transitioning to LTC.
Concept	A transition involves a planned relocation to a LTC facility. Within this review the transitional period is defined as the period of time one begins to consider a permanent relocation to a long‐term care setting until 12 months after the move. This review considered studies which encompassed the range of context and situations where transitions to LTC may occur. These included developmental or life cycle transitions such as ageing which may trigger a relocation of residence (Meleis, 2010: 129); Situational transitions involving relationship or family transitions occurs when one considers or relocates to a long‐term care facility (Davies, 2005), Health‐illness transitions occur within the course of an illness or condition which may impact the person's independence or care requirements (Schumacher & Meleis, 1994). Studies which contained, facilitated or reported on the phenomenon of SDM within the context of transitioning to LTC. The attributes of SDM were based on the conceptual description by Elwyn et al. (2012): Choice talk: Studies which made efforts to ensure that the person or their advocate (family, caregiver or other) understand the reasonable options available. This includes the use of decisional aids, reasonable adjustments, offering choices, preferences, personalised to the person to aid involvement, pros and cons. Option talk: Studies which made efforts to detail available options, checked knowledge, harms/ benefits, supported the person decisions through decisional aids, advocacy or summary. Studies which made efforts to support relational autonomy, namely how decisions can relate to interpersonal relationships and mutual dependencies (Elwyn et al., 2012). Decisional talk: Studies which made efforts to consider the preferences of the person when deciding what is best. Exploring and respecting what matters most to the person leading to informed preferences. The reviewed included studies that contained any of the above attributes and features of SDM.
Context	A LTC residential facility provides a broad range of services including personal, medical or social care which supports people with cognitive or functional limitations to self‐care or other activities (Zimmerman & Sloane, 2007). This scoping review considered such facilities including residential care, assisted living, nursing homes, skilled nursing facilities, continuing care retirement communities where a person resides.
Types of sources	Both qualitative and quantitative studies were considered. Sources include primary research studies, reviews, dissertations and evidence‐based guidelines. Discussion papers, policy documents, commentary, editorials papers were excluded. Grey literature were excluded as this review focuses on peer‐reviewed evidence.

### 2.1 Search strategy

Involved a three step process as prescribed by Peters et al. (2020). Initially a preliminary limiting search of two appropriate databases, ClNAHL and Medline, was undertaken to identify a comprehensive list of relevant text words contained in the title and abstract to refine the search terms. A librarian assisted in further refinement with analysis of MESH headings which informed the development of a full search strategy using all keywords across databases. The full search strategy is outlined in Table 2. The search was inclusive of publications from January 2001 to March 2021, reflective of the emergence of SDM models and approaches within this time period. CINAHL, Medline and Psych Info (EBSCOhost) and Cochrane Review were searched independently. The final step involved bidirectional citation searching of papers included (Hinde & Spackman, 2015). Furthermore, as several protocols were identified from database searches, a detailed search of primary authors' ResearchGate profiles was undertaken.

**TABLE 2:** Search strategy

**Title: Shared decision‐making with adults transitioning to long‐term care: A scoping review 29** ^**TH**^ **March 2021** **CINAHL (EBSCO)** S1 “Care Facilit*” OR “Residential Care” OR “Assisted Living Facilit*” OR “Care Home*” OR “Community Hospital*” OR “Continuing Care” OR “Elder Care” OR “Geriatric Care Facilit*” OR “Gerontolog* Care” OR “Long term care” OR “Long Stay” OR “Nursing Home*” OR “Residential Aged Care Facilit*” OR “Residential Care Home*” OR “Skilled Nursing Facilit*” OR “Supported Care Facilit*” Or “psychogeriatric unit” or “developmental centre” or hous* or residenc* TI or AB. Or using CINAHL headings (MH "Long Term Care") 0r (MH "Residential Care+") or (MH "Residential Facilities+") (181,577) AND S2 Transition* or relocat* or transfer* or mov* or progress* or relinquish* or displacement or resettl* or re‐hous* or rehous* AB & TI OR using CINAHL headings (MH "Transitional Programs") OR (MH "Transitional Care") or (MH "Relocation") (424,244) AND S3 "shared decision making" or "shared decision‐making" or "decision making" or "decision‐making" or "decision making process*" or "decision‐making process*" or "family decision mak*" or "patient decision mak*" or partnership or collaboration or alliance or “goal sharing” or “shared goal*” or “information sharing” or “interprofessional collaboration” or “decisional support*” or “decisional aids” or “decisional coaching” or enablement or “person‐centred*” or “active participation” or “collaborative decision making” or “collaborative partnership” or “collaborative working” or “patient involvement” or “patient participation” or “patient engagement” AB OR TI OR using CINAHL headings (MH "Decision Making, Shared") OR (MH "Decision Making, Organizational") OR (MH "Decision Making, Patient") OR (MH "Decision Making, Family") OR (MH "Decision Making, Clinical") OR (MH "Decision Making, Ethical") OR (MH "Decision Making") (234,426) (Adult as a search term was discussed with librarian I will use the left hand column to include all adults populations at end) SI & S2 & S3 Limiters = English, 2001–2021 & all adult groups ‐ 587. **Medline (EBSCO)** S1 “Care Facilit*” OR “Residential Care” OR “Assisted Living Facilit*” OR “Care Home*” OR “Community Hospital*” OR “Continuing Care” OR “Elder Care” OR “Geriatric Care Facilit*” OR “Gerontolog* Care” OR “Long term care” OR “Long Stay” OR “Nursing Home*” OR “Residential Aged Care Facilit*” OR “Residential Care Home*” OR “Skilled Nursing Facilit*” OR “Supported Care Facilit*” Or “psychogeriatric unit” or “developmental centre” or hous* or residenc* TI or AB. Or using MESH headings OR (MH "Residential Facilities+") OR (MH "Transitional Care") OR (MH "Long‐Term Care") (410,844)
AND S2 Transition* or relocat* or transfer* or mov* or progress* or relinquish* or displacement or resettl* or re‐hous* or rehous* AB OR TI OR using MESH headings **(MH "Transitional Care") OR (MH "Health Facility Moving") OR (MH "Health Transition") OR (MH "Transition to Adult Care")** **(2,744,593)** **And** **S3** "shared decision making" or "shared decision‐making" or "decision making" or "decision‐making" or "decision making process*" or "decision‐making process*" or "family decision mak*" or "patient decision mak*" or partnership or collaboration or alliance or “goal sharing” or “shared goal*” or “information sharing” or “interprofessional collaboration” or “decisional support*” or “decisional aids” or “decisional coaching” or enablement or “person‐centred*” or “active participation” or “collaborative decision making” or “collaborative partnership” or “collaborative working” or “patient involvement” or “patient participation” or “patient engagement” AB OR TI OR using MESH headings (MH "Decision Making, Shared") OR (MH "Decision Making") OR (MH "Clinical Decision‐Making") OR (MH "Decision Making, Organizational") OR (MH "Decision Support Techniques") (371,877) S1 & S2 & S3 = (2080) Limiters: English, 2001–2021’ All adult‐698. **Psych‐info** S1 “Care Facilit*” OR “Residential Care” OR “Assisted Living Facilit*” OR “Care Home*” OR “Community Hospital*” OR “Continuing Care” OR “Elder Care” OR “Geriatric Care Facilit*” OR “Gerontolog* Care” OR “Long term care” OR “Long Stay” OR “Nursing Home*” OR “Residential Aged Care Facilit*” OR “Residential Care Home*” OR “Skilled Nursing Facilit*” OR “Supported Care Facilit*” Or “psychogeriatric unit” or “developmental centre” or hous* or residenc* TI or AB. OR Using APA Thesaurus of psychological index terms DE "Long Term Care" OR DE "Continuum of Care" OR DE "Residential Care Institutions" OR DE "Elder Care" OR DE "Nursing Homes" OR DE "Nursing Home Residents"=30,384 (131,586) And S2 Transition* or relocat* or transfer* or mov* or progress* or relinquish* or displacement or resettl* or re‐hous* or rehous* AB OR TI OR using APA Thesaurus of psychological index terms DE "Transition Planning" OR DE "Life Changes"=5,125 (509,242) And S3 "shared decision making" or "shared decision‐making" or "decision making" or "decision‐making" or "decision making process*" or "decision‐making process*" or "family decision mak*" or "patient decision mak*" or partnership or collaboration or alliance or “goal sharing” or “shared goal*” or “information sharing” or “interprofessional collaboration” or “decisional support*” or “decisional aids” or “decisional coaching” or enablement or “person‐centred*” or “active participation” or “collaborative decision making” or “collaborative partnership” or “collaborative working” or “patient involvement” or “patient participation” or “patient engagement” OR using APA Thesaurus of psychological index terms DE "Patient Centered Care" OR DE "Group Decision Making" OR DE "Decision Making" OR DE "Decision Support Systems" 94,200 (214,439) S1 & S2 & S3 = 1321 Limiters: 2001–20, English & All adult‐ 689

### 2.2 Study selection

Following the full database searches, citations were imported into Covidence software. Source selection at (title/abstract screening and full‐text screening) was performed by two reviewers [CE and MC], independently. The full‐text articles selected for review were considered against the inclusion criteria by the two reviewers with disagreements resolved through discussion. Reasons for exclusions of full text were recorded.

### 2.3 Data extraction

Data were extracted under the following headings author, country, year, aim, definition of transition and SDM, setting, sample, duration, design, results/outcomes and key findings (Table 3).

**TABLE 3:** Data extraction table, categorised by type

Author year and country	Aim	Design duration	Sample	Setting	Definition of SDM	Definition of transition	Results/key findings
**Observational studies**							
Légaré et al. (2014) Canada	Explore the perceptions of family caregivers about the decision‐making process they had experienced. This was regarding the applicability of the IP‐SDM within the context of relocating their relative to LTC and the application of interprofessional approach to shared decision making (IP‐SDM) in this context.	Qualitative exploratory case study Cross sectional	Convenience 6 Family caregivers of an older adult greater 65 years	Community	Yes IP‐SDM	No	Caregivers did not experience IP‐SDM when deciding to relocate a family member to LTC. Resource implications. Lack of options. Limited involvement of the older person. Lack of impartiality of healthcare staff
Garvelink et al. (2019) The Netherlands	Assess the extent that the decision‐making process about housing for people with dementia (PWD) and their caregivers (informal/formal) correspond to the IP‐SDM approach.	Qualitative content analysis. Secondary data analysis of longitudinal multi‐perspective study which examined SDM in care networks. Interviews at 3 different points over 2 years	4 care networks Including 4 older community dwelling people with dementia (PWD) 8 informal caregivers 8 professionals	Community	Yes IP‐SDM	No	Decision‐making within care networks corresponded to SDM, but never included all care network members. Decisions were guided by the PWD but their involvement decreased over time.
Hillcoat‐Nallétamby and Sandani (2019) Wales	Explore how a “moving on” service which facilitates voluntary residential relocations, empowers older people to make informed decision regarding a home from home transition from their current private home to an extra care facility (assisted living).	Qualitative content analysis. Cross‐sectional	Purposeful sampling 18 clients who were recorded to have contacted the “moving on” service and having received at least one in‐person visit or phone call from the service.	Community	No	Yes	Identified 3 patterns of service use continuous, partial and discontinued. Service was instrumental in empowering users to exercise decisional autonomy.

Author year and country	Aim	Design/duration	Sample	Setting	Definition of SDM	Definition of transition	Results/key findings.
**User‐centred designs**							
Lord et al. (2016) UK	A project to create and test a resource to help people with dementia and their family caregivers make decisions about their living arrangements and future place of care.	Qualitative content analysis. Cross sectional	7 (both sexes) individuals with a clinical diagnosis of dementia. (4 carer dyads from sample of 13 family carers) currently in the process of deciding or who had recently decided about, future place of care or residence.	Community	No	No	PWD: Resented not being involved or supported in decision making process, Lack of control/self‐determination/inclusion. Some reported a joint and shared decision between themselves and caregivers. Caregivers: Unbalanced involvement. All carer's and PWD wanted to maintain the person at home. Highlighted the importance of direct professional support Results used to inform the development of a decisional aid.
Garvelink et al. (2016) Canada	Development of a decision aid for use among older adults, their informal caregivers and health professionals about whether to continue living at home or move into residential care.	Iterative user‐centered design with 3 cycles of a paper‐based decisional aid development. Involving the development and refinement of a series of prototypes which were adjusted according to end‐user feedback. Approximately 2 years	(5) informal caregivers who had been previously involved in location of care decisions with their family member. (6) health professionals involved in home‐care delivery. (6) health administrators (experts) (4) Older adult end‐users tested the usability in the final stages.	Community	Yes IP‐SDM	No	Development of an intervention based on potential users needs.
van Leersum et al. (2020) The Netherlands	Understand the user requirements and develop, a web‐based preference elicitation tool for clients in need of long‐term care.	Qualitative descriptive applying a user‐centered design for an iterative process in tool development. Explored the look, feel, navigation and content.	End users who were engaged in decision‐making process for long‐term care or had recently chosen LTC, including service users, relatives and healthcare professionals. Convenience sample of 25 users elderly sector = 2 relatives disability sector = 10 (1 relative 9 clients) mental health = 3 clients social care = 7 clients 3 other 2 healthcare professional and 1 client.	Community	Yes	No	Qualitative results presented under 3 stages, Visual development of intervention, navigation and comprehension
Granbom et al. (2020) Sweden	Develop a prototype of a web‐based housing counselling intervention for later life.	User‐centered design using research circle methodology 7 months Qualitative content analysis	3 different groups of participants. 9 older adults 6 technology and design experts 7 Representatives of companies and non‐profit organisations with knowledge of the housing needs of this population	Community	No	No	“Ageing in the right place” project which developedd a web‐based housing counselling intervention. Involved 3 modules accommodating end users at various stages of decision‐making. Final module involves information on relocating residence
**Experimental designs**							
*Pre and/or post‐test designs*							
Stacey et al. (2014) Canada	Develop a systematic process to create and appraise theory‐based vignettes for illustrating IP‐SDM to health professionals. Vignette scenario: “a client and family member deciding about location of care for a frail elderly person in the process of losing autonomy.	Multi‐phase 6 step process. Retrospective pre/post‐test design to evaluate the video as part of a 3.5 h IP‐SDM skills building workshop 2 months	29 various healthcare professionals who undertook a workshop on IP‐SDM.	Community	Yes IP‐SDM	No	14 min clinical vignette. Workshop was rated as excellent. The vignette was rated as good (20/29), excellent 6(29), weak (3/29). There was a statistical difference in participants self‐report IP‐SDM knowledge after the workshop and increased confidence in using the IP‐SDM. Some qualitative comments indicated that the IP‐SDM video was a good visual tool for learning about the IP‐
Dogba et al. (2020) Canada	Discuss the creation and trial implementation of education tools for an IP‐SDM workshop. Presents the findings of a workshop development process, findings and future IP‐SDM training programme.	Interdisciplinary co‐design of a workshop on IP‐SDM. Evaluation survey Cross‐sectional	219 interdisciplinary health and social care workers	Community	IP‐SDM	No	Confidence using IP approach when supporting seniors make decisions whether to remain at home or move to residential care. Majority rated workshop as excellent, vignette presentation and group discussion excellent.

Author year and country	Aim	Design/duration	Sample	Setting	Definition of SDM	Definition of transition	Results/key findings. Outcomes
**Randomised controlled trials**							
Mukamel et al. (2016) USA	Test whether the NHCPlus embedded in a discharge can lead to better outcomes than the usual process of discharge from hospitals to nursing homes. A web‐based app designed to improve the decision‐making process for families and patients transitioning to a nursing home from an acute care setting	Evaluation of an intervention using a 2 armed RCT. Quantitative 18 months	225 patients admitted to the hospital from the community and discharged to a nursing home.	Hospital setting	No	No	About 85 percent of users indicated satisfaction with NHCPlus. Compared to controls, intervention patients were more satisfied with the choice pro‐ cess (by 40 percent) Findings demonstrate that they lead to greater patient confidence and satisfaction; higher probability of discharge to better quality nursing homes based on two indicators of quality:

Author year and country	Aim	Design/duration	Sample	Setting	Definition of SDM	Definition of transition	Outcome measures
**Protocols**							
Légaré et al. (2015) Canada	To evaluate the impact of training interprofessional home care teams in SDM combined with a decisional aid on the proportion of elderly people who report being active in the decision‐making process regarding whether to stay at home or move to a care facility.	Propose a multi‐centred cluster RCT among homecare IP teams with 2 data collection before and after. Pre (3 months) and post‐test (10–12 months) intervention. Intervention arm: Training in IP‐SDM, decision aid Control group: usual care Quantitative	Cluster size 12‐16. Estimation: 501 clients included Clients >65 years or Informal Caregivers receiving care from the Interprofessional at home. Have faced a decision about whether to stay or move to care facility in the previous 3 months.	Community	IP‐SDM	No	Primary Proportion of elderly people who report an active role in decision making. Controlled preference's scale Secondary: Preferred health related housing option and actual health related housing decision, Decisional conflict scale, decision regret scale, Zarit burden interview.
Légaré et al. (2016) Canada	Evaluate the impact of a training programme in IP‐SDM on the proportion of clients who report taking an active part in decision‐making compared with passive dissemination of a decision guide. One question which the trial addresses whether to stay at home or move to another location.	Stepped wedge cluster randomised trial to evaluate intervention involving 8 HSSC (Health and social care centres) and an IP teams from each. HSSC will be randomised to 1 of 4 steps intervention start time separated by 7 months intervals. Data will be collected on different (cross‐sectional) samples of clients and caregivers at each collection point. Quantitative	IP Homecare teams within HSSC. Clients >65 years and Informal caregivers receiving care. Have faced a decision about whether to stay or move to another location during the recruitment period. Est: 320 clients and 320 caregivers	Community	IP‐SDM	No.	Primary outcome clients and caregivers assumed role in decision making. Controlled preference's scale modified for the older adult. Secondary: D‐OPTION scale SDM behaviours during decision making. Preferred and chosen options (remain at home or move), Uptake of decision guide, Health related QOL, Decisional conflict scale, decision regret scale, Zarit burden interview (caregivers). Healthcare professionals intention to engage in SDM, before and after intervention. Evaluation of workshop and tutorial Qualitative field notes from research assistants engaging with clients and caregivers.

Author year and country	Aim	Design/duration	Sample	Setting	Definition of SDM	Definition of transition	Results
**Evaluations of experimental papers**							
Boucher et al. (2019) Canada	Explored factors associated with burden of care among informal caregivers who had made a housing decision on behalf of a cognitively impaired older person.	Secondary data analysis from a RCT (Légaré et al., 2015) Quantitative (descriptive and multi‐level modelling) Primary Outcome Measure: Caregiver burden of care Independent variables: Decision regret, Decisional conflict, control preference scale, C‐OPTION, SDMQ‐9	296 Informal caregivers involved in making a housing decision on behalf of a cognitively impaired older adult >65 years receiving care from the interprofessional home care team. The sample included those from both the intervention and control group of the RCT.	Community	Yes‐ IP‐SDM	No	Caregivers who experienced higher burden were female, higher decision regret and decisional conflict. Caregivers' wo perceived that a joint decision‐making process had been made experienced higher burden

Author year and country	Aim	Design/duration	Sample	Setting	Definition of SDM	Definition of transition	Outcome measures
Adekpedjou et al. (2020) Canada	Assessed the effect of training homecare teams in IP‐SDM on caregivers who reported to be active in decision‐making. This is regarding health related housing for a cognitively impaired older adult.	Secondary data analysis from a RCT (Légaré et al., 2015) Quantitative (descriptive and multi‐level modelling) Primary Outcome Measures: Control preference scale Secondary: Preferred health related housing option and actual health related housing decision, Decisional conflict scale, decision regret scale, Zarit burden interview.	Control group: 165 caregivers 130 health care professionals Intervention group: 144 Caregivers 122 healthcare professional Who received the IP‐SDM training.	Community	Yes IP‐SDM	No	Intervention increased the proportion of caregivers who reported taking an active role in decision‐making between 12–18 per cent. Preferred role and their actual role was a greater match. Intervention showed no effect on secondary outcomes making no difference between preferred option and decision made.

Author year and country	Aim	Design/duration	Definition of SDM	Definition of transition	Findings
**Literature reviews**					
Gravolin et al. (2007)	To assess the effects of various decision‐making support interventions delivered by health or social care providers on the outcomes of older people facing the possibility of entering LT residential care.	Systematic Review	No	No	No papers met the inclusion criteria.
Manthorpe and Martineau (2010)	To identify and analyze research findings in the area of advocacy around care home entry.	Scoping review	No	No	Neglect of the subject. None of the reports and articles included directly focused on advocacy and entry into a care home.

Author year and country	Aim	Design/duration	Definition of SDM	Definition of transition	Themes
**Evidence‐based practice guidance**					
Hertz et al. (2016)	Evidence‐based practice guideline focused on management of relocation in cognitively intact older adults.	Practice guidance	No	Yes	Pre and post relocation guidance for assessment and interventions.

Author, year and country	Aim	Design/duration	Sample	Setting	Definition of SDM	Definition of transition	Results
**Development of a transitional model**							
Groenvynck et al. (2021) The Netherlands	Proposes a model to optimise transitions care from home to a nursing home.	Literature review. The review results were mapped onto pre, mid or post transition phases to create the TRANSCIT model. Preliminary validation of model	Validation = Academic and research professionals. 16 experts in practice	Academic	No	Yes. Identified 3 stages pre, mid or post transition	The model identified 4 components support, information, communication and time identifying an overall need for partnership during transitions. The model presented clear practical examples of the 4 components.

### 2.4 Analysis of the evidence and presentation of results

The scoping review is reported narratively using a combination of the Peters et al. (2020) framework for scoping review and the PAGER Framework (Bradbury‐Jones et al., 2021). The PAGER approach was chosen as it details a consistent approach to charting and synthesis which the PRISMA (Tricco et al., 2018) extension of scoping reviews omits (Bradbury‐Jones et al., 2021).

## 3 RESULTS

**FIGURE 1:**
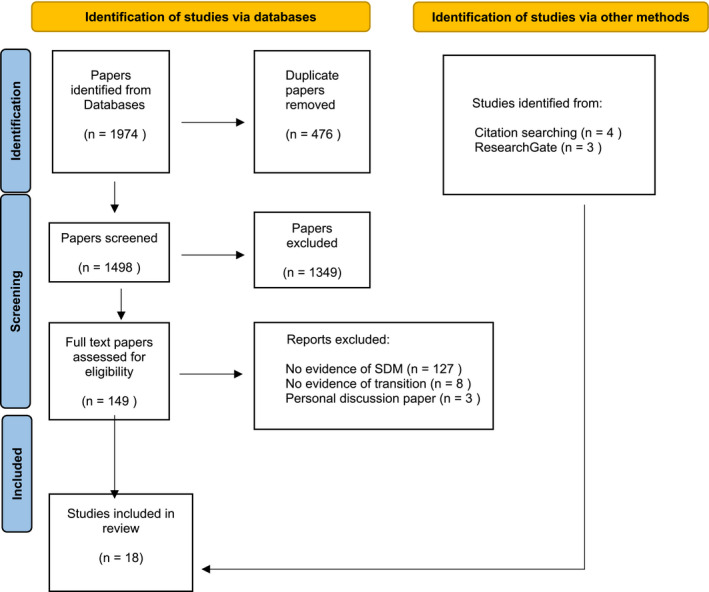
Prisma Flow chart

The search identified 1974 papers with 476 duplicates removed. There were 1498 papers screened by title and abstract, from which 1349 papers were deemed irrelevant. The remaining 149 papers were read in full and, 127 did not meet the inclusion criteria. Citation searches (*N* = 4) and first authors searches on ResearchGate (*N* = 3) identified an additional seven papers which met the inclusion criteria. In total, 18 papers were included in this review. The search is reported as per PRISMA flowchart Figure 1.

The results were aligned and synthesised according to the PAGER framework detailed in Table 4.

**TABLE 4:** Pager framework

Patterns	Advances	Gaps	Research recommendations	Evidence for practice
Advancements in SDM during transitions to LTC. Framing the concept The emergence of interventions Theoretical underpinnings Barriers to SDM during transitions to LTC Facilitators to SDM during transitions to LTC	Evidence from the Canadian homecare system involving an interprofessional model. Evidence of microlevel engagement Evidence involving samples of older adult. Evidence of advances in both the evidence‐base and methodological approaches from observational to experimental designs. Evidence of theoretical developments. There are advancements in user‐centred iterative designs. There is evidence of proactive supports which accommodate individuals at various stages of decision‐making. There is growing evaluations from a Canadian clinical trial relating to the impact of decisional‐support interventions among family caregivers. There is evidence of a lack of inclusion of older adults in SDM during transitions to LTC. There is evidence of the impact resources has upon SDM within the context of transitioning. Evidence also exists of supporting one's ability to exercise choice through SDM. Evidence of collaboration between the older adult, family caregivers and healthcare professionals working together to support decision‐making during transitioning to LTC	There is a deficiency of diversity among sampling strategies. At the time of this review, the search identified no evidence from the older adults perspective from the Canadian trials (Légaré et al. 2015, 2016). No evidence of longitudinal research. There is a need to address the acceptability and useability of interventions among adults who are not proficient with technology and the Internet.	There is a need to explore SDM among diverse populations who may be faced with a transitional decision. To carry out both observational and experimental participatory research on diverse populations and socio‐demographic groups. Explore the integration of a transitional model into future research and practice. Research into macrolevel engagement. Research into training professionals who are supporting other vulnerable or cross‐cultural/demographic populations who may be experiencing a transition towards LTC. Longitudinal research into the effectiveness of IPSDM over time and at different transitional points in the person and their caregivers' journey. Future research focusing on qualitative or mixed method designs to explore experiences of IPSDM in practice is warranted. A future systematic review when there is sufficient evidence from interventional studies is recommended to evaluate the effectiveness of interventions. Research community agreement on core standardised outcome measures at patient, carer, staff and organisational level to allow comparison and meta‐analysis.	The evidence reflects the interprofessional and interdisciplinary teams of clinicians, researchers, policy makers, research commissioners and service providers who supports these adult populations in practice environments. The theoretical model IP‐SDM facilitates clear practical examples of SDM to inform practice with tested user friendly resources The evidence from this review reflects the individual's right to self‐determine. Interventions reflect legal and care‐ethical approaches to practice. Providing evidence of person‐centred approaches. It is important that practitioners consider individual family contexts and resource availability when engaging in SDM within the context of transitioning. The evidence alluded to the importance of a involving a professional and counselling to complement and support decisional aids in practice.

### 3.1 Characteristics of included papers

The papers were mainly published between 2014 and 2021 reflecting recent interest in this area. Before this period, there is a dearth of evidence which was captured in the two included reviews the search identified (Gravolin et al., 2007; Manthorpe & Martineau, 2010). Geographically, the majority of the papers (*N* = 8) originated from the Canadian homecare setting, with the Netherlands (*N* = 3), UK (*N* = 3), the United States (*N* = 1), Australia (*N* = 1) and Sweden (*N* = 1) also contributing to the knowledge base. There has been an expansion in interest among interprofessional services (*N* = 9). The evidence was at microlevel involving interprofessionals in day to day practice engaged in small scale research studies over short durations. Patterns in sampling largely focused on older adult populations (*N*−17); however, the population descriptors did not always make clear the cognitive capability of people transitioning to LTC. Seventeen of the papers were community based transitions from the participants' homes to LTC with the final paper involved a transition from an acute setting to LTC (Mukamel et al., 2016). There were a wide range of designs included, which are discussed later under advances in SDM during transitions to LTC. The characteristics of included papers are detailed in Table 5.

**TABLE 5:** Characteristics of included studies

Country of origin	Papers
Canada (8)	Adekpedjou et al., 2020; Boucher et al., 2019; Dogba et al., 2020; Garvelink et al., 2016; Légaré et al., 2015, 2016, 2014; Stacey et al., 2014
The Netherlands (3)	Garvelink et al., 2019; Groenvynck et al., 2021; van Leersum et al., 2020
Sweden (1)	Granbom et al., 2020
US (2)	Mukamel et al., 2016; Hertz et al., 2016
Austrailia (1)	Gravolin et al., 2007
UK (3)	Hillcoat‐Nallétamby & Sandani, 2019; Lord et al., 2016; Manthorpe & Martineau, 2010.

Discipline	
Inter‐professional (IP) home care workers = direct care staff (9)	Adekpedjou et al., 2020; Boucher et al., 2019; Dogba et al., 2020; Garvelink et al., 2016, 2019; Légaré et al., 2015, 2016, 2014; Stacey et al., 2014
Nurses (1)	Hertz et al., 2016
Independent care coordinator (2)	Hillcoat‐Nallétamby & Sandani, 2019; Van Leersum et al., 2020
Not specified (6)	Gravolin et al., 2007; Granbom et al., 2020; Groenvynck et al., 2021; Lord et al., 2016; Mukamel et al., 2016; Manthorpe & Martineau, 2010.

Design	Number of papers	Author and design
Observational	3	Légaré et al. (2014) qualitative exploratory Garvelink et al. (2016) qualitative content analysis Hillcoat‐Nallétamby and Sandani (2019) qualitative content analysis
User‐centered designs	**4**	Lord et al. (2016) Qualitative content analysis. van Leersum et al. (2020), User centered design and development of an intervention. Granbom et al. (2020), User centered design of an intervention prototype. Garvelink et al. (2016) User centered design and development of an intervention.
Experimental pre and post‐test	**2**	Stacey et al. (2014) Creation and test of an intervention with post measures. Dogba et al. (2020) Evaluation survey of intervention
RCT	**1**	Mukamel et al. (2016) RCT controlled before and after intervention study
Protocols	**2**	Légaré et al. (2016) RCT Légaré et al. (2015) RCT
Evaluations	**2**	Boucher et al. (2019) Secondary data analysis from RCT. Adekpedjou et al. (2020) Data analysis from RCT
Reviews	**2**	Gravolin et al. (2007) Systematic review Manthorpe and Martineau (2010) Scoping review
Evidence‐based Practice guideline	1	Hertz et al. (2016)
Development of a Transitional model	1	Groenvynck et al. (2021)

Intervention	Author
Paper‐based decisional aids/guides	Garvelink et al. (2016), Lord et al. (2016)
Electronic/Web‐based decision aid	Granbom et al. (2020); Mukamel et al. (2016), van Leersum et al. (2020)
Clinical vignette	Stacey et al. (2014)
IP‐SDM training for homecare staff Some integrating a DA	Légaré et al. (2015) Dogba et al. (2020) Légaré et al. (2016)

The review identified a diverse body of knowledge on SDM during transitions to LTC. Three overall patterns were identified: advances and innovation in SDM during transitions to LTC, facilitators of SDM during transitions to LTC, and barriers to SDM within this context.

### 3.2 Advances in SDM during transitions to LTC

Advances represent the developments and innovation in both the evidence‐base and methodological approaches to research exploring SDM and transitioning to LTC. The sub‐patterns of framing the concept, theoretical advancements and the emergence of interventions illustrate advances in operationalising SDM into practice.

#### 3.2.1 Sub‐pattern 1: framing the concept

The observational papers reflect an aspiration to gain insight into the experiences of SDM among caregivers and their family members during transitions to LTC (Garvelink et al., 2019; Hillcoat‐Nallétamby & Sandani, 2019; Légaré et al., 2014). Légaré et al. (2014) and Garvelink et al. (2019) explored the experiences and extent that participants were involved in SDM in housing decisions. Légaré et al. (2014) included caregivers who faced a decision whether their family member should remain at home or move to a LTC facility. Garvelink et al. (2019) further advanced the knowledge base by exploring SDM among people with dementia and their family caregivers at three time points representing key transitional periods. Both Légaré et al. (2014) and Garvelink et al. (2019) benchmarked their findings against a model which facilitated SDM during a transitional period involving a possible relocation to a LTC facility. They concluded that caregivers and the older person attitudes to and experiences of SDM during transitioning were valued but proved challenging to operationalise in practice. Recognising the tension which exists between their ideal and actual experience of SDM. These qualitative findings recognised the importance of involving the older person to ensure that the evidence base reflects their voice. Researchers acknowledged that to advocate for SDM, it is imperative that the end‐users are involved in designing research, thus ushering in an era of co‐design and user‐centred designs.

#### 3.2.2 Sub‐pattern 2: emergence of interventions

The first intervention papers included user‐centred iterative designs (Garvelink et al., 2016; Granbom et al., 2020; Lord et al., 2016; van Leersum et al., 2020), representing advancement in how the topic was approached. Qualitative and quantitative descriptive findings were used to inform the development of decisional supports (Garvelink et al., 2016; Granbom et al., 2020; Lord et al., 2016; van Leersum et al., 2020). Earlier papers (Garvelink et al., 2016; Lord et al., 2016) developed paper‐based decisional aids as a resource to help participants with decisions about moving into residential care, which were designed to present choice and avoid over directing decisions. Subsequent papers (Granbom et al., 2020; van Leersum et al., 2020) embraced web‐based or electronic formats including a web‐based housing counselling service and a web‐based preference elicitation tool.

User‐centred designs have evolved to embrace technology. Furthermore, interventions recognised that SDM must involve a choice between relocating residence or to age in place. Reflecting advancements in co‐design subsequent papers aimed to establish intervention fidelity through experimental designs representing more sophisticated attempts to measure the developments and impact of research into SDM and transitioning to LTC.

Experimental research has been a feature of Canadian papers. Both Stacey et al. (2014) and Dogba et al. (2020) developed training material and evaluated an educational intervention involving an IP‐SDM (Interprofessional shared decision‐making) program which involved a homecare team and an older person in a decision on whether to remain at home or move to a LTC facility. These interventions were implemented as part of the design in two related study protocols: a multi‐centre cluster RCT (Légaré et al., 2015) and a stepped wedge cluster RCT (Légaré et al., 2016) which aimed to evaluate the impact of an IP‐SDM training among interprofessional staff, caregivers and older adults compared to the control group receiving usual care.

From this Canadian trial, there are two recent publications which report on family caregivers experiences (Boucher et al., 2019) and (Adekpedjou et al., 2020). Boucher et al. (2019) primarily measured burden of care and how this was influenced by decisional regret, conflict and preference among family caregivers (*n* = 296) who had received support from a homecare staff trained in IP‐SDM and had made a housing decision on behalf of a cognitively impaired older person. The results illustrate that concepts, such as burden, were often reflective of how caregiver's felt when they tried to honour the preferences and values of their loved one. Adekpedjou et al. (2020) reported that caregivers (*n* = 309) who perceived an active role in decision‐making regarding housing for a cognitively impaired older adult using a control preference scale increased from 12 to 18 per cent for the intervention group. Secondary outcome measures included preferred versus actual housing option, decisional conflict, regret and burden showed no effect upon caregivers.

#### 3.2.3 Sub‐pattern 3: theoretical underpinnings

The review also maps patterns in theoretical developments. The majority of papers (Adekpedjou et al., 2020; Boucher et al., 2019; Dogba et al., 2020; Garvelink et al., 2016, 2019; Légaré et al., 2014, 2015, 2016; Stacey et al., 2014) integrated a theoretical framework IP‐SDM as defined by Légaré et al. (2011). This is a process by which health related decisions are made jointly by a client and his/her health professional based on the available evidence and what matters most to the person which is used to inform an agreed upon decision (Légaré et al., 2014). Additionally, van Leersum et al. (2020) utilised the Elwyn et al. (2012) collaborative deliberation model as a conceptual model.

Within the majority of papers, the concept of transition was a contextual element and SDM was explored in respect to decisions in terms of planning, moving or relocating to LTC. There is a lack of attention to developing and integrating theoretical and conceptual frameworks on transitioning in the evidence. Indeed, Hertz et al. (2016); Hillcoat‐Nallétamby and Sandani (2019); and Groenvynck et al. (2021) were the only papers to define transition. Most of papers focused on pre and during transitional decision‐making except Groenvynck et al. (2021) who proposed a transitional model which identified practice‐based supports and approaches during the pre‐, mid‐, and post‐transitional period to reflect the characteristics of SDM. This study proposed to optimise transitional care for older adults and their caregivers; however, the model has yet to be integrated and evaluated in research and practice.

### 3.3 Facilitators to SDM during transitions to LTC

The review identified enabling factors including proactive planning, exercising choice and collaboration as key to supporting the person and their family caregiver(s) to make decisions about their living arrangements and future place of care (Garvelink et al., 2019; Hillcoat‐Nallétamby & Sandani, 2019; Légaré et al., 2014; Lord et al., 2016). The evidence suggests that practices which enable the person's involvement in decision‐making must be flexible to respond to emerging needs and fluctuating capacity (Lord et al., 2016). Involvement of family caregivers was viewed as a facilitator of SDM, as their decisions strongly reflected the wishes and preferences of the person and demonstrated a respect for their voice which was of great importance to family caregivers (Garvelink et al., 2019). Proactive planning for future housing needs also facilitated SDM, recognising that decisions and preferences should be elicited earlier when self‐determination may be easier (Granbom et al., 2020; Hertz et al., 2016; Hillcoat‐Nallétamby & Sandani, 2019).

Supporting one's ability to exercise choice is key to SDM. Lord et al. (2016) reported that enabling SDM among older adult populations involved providing several options such as home‐based supports, rather than relocating to LTC as the only option. Subsequent SDM designs involved decisional supports about whether to remain at home or move to a LTC setting (Garvelink et al., 2016; Granbom et al., 2020; Légaré et al., Légaré et al., 2015, 2016). This integrated both relocation and ageing in place as choices allowing users to weigh‐up the benefits and drawbacks personalised to their individual situation.

Collaboration between the older adult, family caregivers and healthcare professionals working together to support decision‐making during transitioning to LTC was identified as fundamental to facilitate SDM (Groenvynck et al., 2021; Hillcoat‐Nallétamby & Sandani, 2019; Lord et al., 2016). Such partnerships aim to crystalise decisions, exchange information, elicit preferences and their feasibility which is reflective of the IP‐SDM model (Légaré et al., 2014). However, the evidence on professionals supporting SDM during transitioning is not consistent, Légaré et al. (2014) caregivers' did not experience IP‐SDM when deciding to relocate a family member to LTC with decisions tinged with pressure and a lack of interprofessional support noting a lack of collaboration between the person, their caregivers and homecare professionals.

### 3.4 Barriers to SDM during transitions to LTC

The papers identified consistent barriers namely a lack of inclusion of the person and disparities between the persons' wishes and the availability of resources (Garvelink et al., 2019; Légaré et al., 2014; Lord et al., 2016). A recurring narrative was that adults did not feel involved and supported in decision‐making with family members often making the decision (Garvelink et al., 2019; Lord et al., 2016). Conversely, family caregivers were aware that they were excluding the person; however, they felt the situation necessitated a move which the person lacked insight or did not want to acknowledge (Garvelink et al., 2019; Lord et al., 2016). In Canada, Légaré et al. (2014) explored this phenomenon focusing on family caregivers with participants reporting a lack of IP‐SDM whereby their values, preferences and that of their family members were difficult to reconcile into shared decisions. Caregivers' highlighted a lack of information and options, noting that the availability of resources and time strongly influenced decision‐making (Légaré et al., 2014). Similarly, Garvelink et al. (2019) reported that as cognitive capability declined due to dementia the person's involvement in decision‐making decreased as SDM was strongly influenced by cognitive functioning. Furthermore, a transitional decision became more likely and family caregivers became more involved with cognitive decline (Lord et al., 2016; Garvelink et al., 2019). Individual family circumstances, practicalities and feelings of stress among caregivers were identified as further barriers which influenced their willingness and motivation to continue caring or engage in SDM (Garvelink et al., 2019; Lord et al., 2016).

The papers have identified the complicated nature of such decision‐making identifying that a stand‐alone decisional aid may be of limited value compared to multi‐pronged interventions (Garvelink et al., 2016; Lord et al., 2016). Approaches which involve professionals and counselling support to complement such decisional aids (Groenvynck et al., 2021; Lord et al., 2016; Hillcoat‐Nalletamby & Sandari, 2019) were valued noting the importance of human contact. Furthermore, concerns were identified regarding web‐based designs for adults who were not proficient with technology and the Internet (Granbom et al., 2020; Mukamel et al., 2016).

**FIGURE 2:**
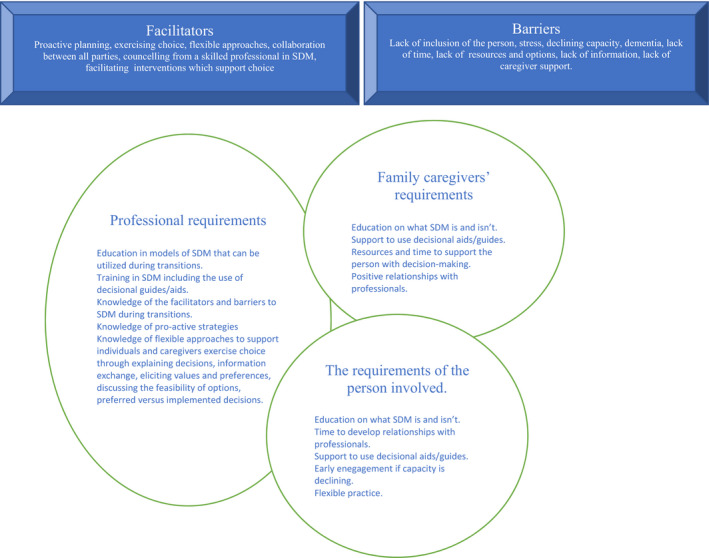
Schematic of the barriers, faciliatators and requirements to facilitate SDM with adults transitioning to LTC

A schematic of the barriers, facilitators and requirements to facilitate SDM with adults transitioning to LTC is presented in Figure 2.

## 4 DISCUSSION

This scoping review has identified advances in theory, methodological approaches and the evidence‐base from observational papers which developed knowledge of adults' experiences of SDM during LTC transitions to user‐informed experimental designs evaluating interventions. Moreover, the results identified the facilitators and barriers to SDM during transitions of this nature.

The overall corpus of literature acknowledges that much of the evidence on SDM in the context of transitions to LTC is in its infancy with the phenomenon only recently receiving attention. The prominence of evidence from the Canadian perspective may be positively influenced by a funding environment supportive of Interprofessional SDM models (Härter et al., 2017). However, caution must be noted as much of the evidence is closely linked to the Canadian homecare system and may not be readily transferable to other healthcare systems or policy.

The perspectives identified in the literature were at microlevel involving older adults, their caregivers and professionals. There is an absence of evidence from the macrolevel involving organisation, governmental, policy direction and how decisions and engagement at this level influence healthcare provision and resources. Macrolevel engagement is essential for SDM to be sustained and entrenched in legislation, regulations and practice through the provision of ongoing resources and organisational drivers (Elwyn et al., 2013; McCafferty et al., 2011; Scholl et al., 2018). Indeed, several of the barriers to SDM identified in this review such as inadequate resources require macrolevel strategies to address. Despite the growing body of evidence, there continues to be limited engagement at governmental and policy level.

The review identified several challenges to SDM during transitions including a lack of inclusion of the person especially in the context of declining cognitive capability. Caregivers were aware that the situation necessitated a move with which the person with dementia disagreed (Lord et al., 2016). Moreover, if the person with dementia has a negative view of LTC placement caregivers may feel compelled to make the necessary decisions without them (Ducharme et al., 2012; Miller et al., 2016). Such challenges may result in caregivers going against their values and preferences causing dissonance. Koenig et al. (2014) identified both congruent and dissonant narratives between older adult and their caregivers when examining their joint experience of this transitional process. Furthermore, cultural traditions such as filial piety (Chen, 2015) may present additional challenges to decision‐making and by association efforts to engage in SDM approaches.

In practice settings, the appropriateness and timing of such interventions to support decision‐making must be critically evaluated by nurses and other professionals on a case‐by‐case basis cognizant of challenges which individuals and their families may experience. It is questionable whether existing SDM models and interventions are suitable to facilitate decision‐making from this perspective. In effect differing perspectives reflect the nuanced and varied challenges which SDM presents across different specialities and individual circumstances (Kalsi et al., 2019). There are also resource and economic implications for integrating these interventions into practice including adequate access to actual or alternative healthcare services to put SDM into practice (Gravel et al., 2006).

The evidence raises awareness of how we involve individuals in transitional decision‐making. There is a moral and ethical impetus on professionals including nurses (American Nurses Association, 2015) and society to include the person in such decisions reflecting a respect for the person's autonomy and right to self‐determine. Such evidence aligns with the United Nations sustainable developmental goals (United Nations, 2015) number 10 and 16 namely to address discrimination, inequality and the inclusion of all populations. Internationally, countries are recognising and legislating for the person's right to self‐determine through supported decision‐making rights and law (Assisted Decision‐Making Capacity Act, 2015; Mental Capacity Act, 2005; United Nations, 2006). This places a responsibility on society to support decision‐making both from a legal and care‐ethical approach enabling the individual to exercise their legal capacity to the greatest extent according to their wishes. Indeed, research in this area reflects societies respect for an individual's right to self‐determine.

### 4.1 Gaps and future research recommendations

The PAGER framework highlights several gaps and future research recommendations. Légaré et al. (2015; 2016) proposed measuring the proportion of older adults who report an active role in decision‐making about whether they remain at home or move to a LTC facility. At the time of this review, the author is not aware of any published papers evaluating older adults experiences. Such evidence on the effect of IP‐SDM is important to inform future research while identifying contextual factors which impact effectiveness among different cohorts of adults. There is a lack of diversity among the sampling strategies employed with a paucity of evidence relating to other younger cohorts who may also become involved in a transitional decision.

The literature review has identified the need for longitudinal and qualitative research into IP‐SDM. Concerns regarding web‐based designs for adults who are not proficient with technology were identified (Granbom et al., 2020; Mukamel et al., 2016). There is a risk that a considerable number of eligible populations would be excluded from these interventions. Therefore, there is a need to address the acceptability and useability of interventions among populations.

A future systematic review when there is sufficient evidence from interventional papers is recommended to evaluate the effectiveness of interventions. There is also a need to agree core standardised outcome measures at patient, carer, professional and organisational level to allow comparison and meta‐analysis.

### 4.2 Evidence for practice

In consideration of the predicted increase in health and social care staff who will be supporting society (OECD, 2022) attention to their education and training needs is warranted. This review sheds light on evidence which may inform training and practice among community gerontological nurses, other professionals' and care providers supporting adults and their caregivers with transitional decision‐making. The interdisciplinary focus of the review reflects interprofessional and interdisciplinary teams of clinicians, researchers, policy makers, research commissioners and service providers who supports these adult populations. The development of the theoretical model IP‐SDM facilitates clear practical examples of SDM to inform gerontological nursing practice (Dogba et al., 2020; Stacey et al., 2014).

### 4.3 Strengths/limitations

A strength of the design is the adherence to a systematic and replicable framework to evidence sourcing, selection, extraction and analysis following the PRISMA extension of scoping reviews framework (Peters et al., 2020). The application of the PAGER framework (Bradbury‐Jones et al., 2021) facilitated the identification of advances in this field, providing further justification for how the gaps and research recommendations were mapped.

There were limitations in time and resources; therefore, grey literature was excluded with the review focused on peer reviewed literature. A further limitation was the limited availability of relevant literature and the inclusion of protocols which reflects the recent emergence of evidence in this area. The authors used collateral strategies, for example searching ResearchGate network to identified relevant resources.

## 5 CONCLUSION

This review has identified and documented the nature and extent of empirical literature related to SDM during transitions to LTC settings. The evidence originates from a variety of scientific fields with an interprofessional focus. The heterogeneity in design and approaches reflect methodological developments from inceptual observational papers, to robust fidelity trials and theoretical advancements. The findings offer evidence of enablers and barriers experienced by the person, their family, nurses and other professional caregivers who were engaged in SDM. Moreover, it identified the complicated and nuanced nature of such decision‐making. In essence, this review illustrates a societal aspiration for protecting a persons' right to be central in all decisions regarding their life. Yet, efforts to engage in SDM during transitions are constrained by the availability of resources. The review highlights gaps in knowledge especially in relation to the inclusion of the person at the centre of the decision as well as culturally specific resources and training. The review highlights approaches that can inform future practice and research to support all adults who may face a transitional decision to actively participate in SDM to their desired degree.

## CONFLICT OF INTEREST

The author(s) declare none.

## Data Availability

The data that support the findings of this study are available in the supplementary material of this article.
